# Hypogammaglobulinemia: a diagnosis that must not be overlooked

**DOI:** 10.1590/1414-431X20198926

**Published:** 2019-10-10

**Authors:** F.M.C.A. Pimenta, S.M.U. Palma, R.N. Constantino-Silva, A.S. Grumach

**Affiliations:** 1 Pós-graduação em Ciências da Saúde, Faculdade de Medicina do ABC (FMABC), Santo André, SP, Brasil; 2 Departamento de Pediatria, Faculdade de Medicina do ABC (FMABC), Santo André, SP, Brasil; 3 Laboratório de Imunologia Clínica, Faculdade de Medicina do ABC (FMABC), Santo André, SP, Brasil; 4 Disciplina de Imunologia Clínica, Faculdade de Medicina do ABC (FMABC), Santo André, SP, Brasil

**Keywords:** Primary immunodeficiency, Common variable immunodeficiency, Hypogammaglobulinemia, Chemotherapy, Antibody defects, Immunoglobulin therapy

## Abstract

Humoral immunological defects are frequent and important causes of hypogammaglobulinemia, leading to recurrent infections, autoimmunity, allergies, and neoplasias. Usually, its onset occurs in childhood or during the second and third decades of life; however, the diagnosis is made, on average, 6 to 7 years afterwards. As a consequence, antibody defects can lead to sequelae. Here we describe the clinical-laboratory characteristics, treatment, and prognoses of patients with hypogammaglobulinemia. An observational, cross-sectional, and retrospective study of patients attending the recently established outpatient group of Clinical Immunology between 2013 and 2018 was carried out. Patients with IgG levels below 2 standard deviations from the mean values for the age and/or impaired antibody response were included. Eight patients (3 F and 5 M; median age=41 years (16–65), average symptom onset at 25 years (1–59), and time to diagnosis of 10 years were included. The main infections were: sinusitis in 7/8, pneumonia in 6/8, otitis in 2/8, tonsillitis and diarrhea in 2/8, and diarrhea in 2/8 patients. Hypothyroidism was identified in 4/8 (50%) patients. Rhinitis was found in 7/8 (87.5%) and asthma in 3/8 (37.5%) patients. The tomographic findings were consolidations, atelectasis, emphysema, ground glass opacity, budding tree, bronchial thickening, and bronchiectasis. Immunoglobulin reposition was used between 466 and 600 mg/kg monthly (514.3 mg·kg^-1^·dose^-1^). Prophylactic antibiotic therapy was included in 7/8 (87.5%) patients. Airway manifestations prevailed in patients with hypogammaglobulinemia. There is a need for educational work to reduce the time of diagnosis and initiation of treatment, avoiding sequelae.

## Introduction

Primary immunodeficiencies (PIDs) represent a group of approximately 350 diseases resulting from an intrinsic immune system defect (1). In general, they are hereditary monogenic diseases that can manifest in the form of increased susceptibility to infections, severe allergies, tumors, or autoimmune diseases (2). The most recent classification of these immunological defects includes disturbances of immune regulation, autoinflammatory diseases, and even phenocopies that mimic the clinical manifestations of immunodeficiencies. Thus, a broader concept of immune response disorders was created and termed Inborn Errors of Immunity (1). Humoral immunodeficiencies (involving antibody defects) are the most frequent and are responsible for more than half of all cases (3).

Hypogammaglobulinemias are heterogeneous diseases of either primary origin (genetic disorders and/or chromosome anomalies) or secondary origin (induced by extrinsic factors – infectious agents, mediators such as corticosteroids and immunosuppressants, chemotherapy, metabolic diseases such as nephrotic syndrome, nutritional disorders, and environmental conditions such as ionizing radiation) (4). Among the conditions associated with hypogammaglobulinemia, common variable immunodeficiency (CVI) is the most common disorder associated with antibody deficiency and has a notably high prevalence, affecting one in every 10,000–50,000 live births.

Infectious processes affecting the airways comprise the main manifestations of PIDs, and are characterized by recurrent conditions, that is, at a rate higher than the average for their age group and may be caused by unusual pathogens (5,6). To improve the recognition of PIDs, 10 warning signs have been developed by the Jeffrey Modell Foundation and the American Red Cross. These signs represent a milestone for improved identification of suspected patients, resulting in earlier diagnoses and, consequently, reducing morbidity and mortality (7). In Brazil, 77% of physicians who participated in a recent survey were not familiar with the warning signs of PIDs (8). Increasing the medical community's awareness of these diseases is an important step towards improving their recognition and treatment. A delayed diagnosis results in increased morbidity and mortality (9), clearly observed in respiratory tract cases, regardless of the immunological defect.

Considering the relevance of early diagnosis and the performance of the specialist in these cases, the present study aimed to describe the diagnosis of patients with hypogammaglobulinemia in a newly-created referral service.

## Material and Methods

An observational, cross-sectional, and retrospective study was carried out at the Outpatient Clinic of Clinical Immunology at ABC University Health Center, which analyzed the medical records of patients with hypogammaglobulinemia between 2013 and 2018. The study was approved by the Research Ethics Committee of the ABC Medical School (CAAE 34466214.9.0000.0082), and written informed consent was obtained from all participants and/or guardians.

The diagnostic criteria for hypogammaglobulinemia followed those established by the European Society of Immunodeficiency (ESID) (10). A marked decrease in IgG concentrations (at least 2 standard deviations below the mean for the age group) and reduction in serum levels of at least one of the IgM or IgA isotypes were considered, in addition to complying with the following criteria: a) age above two years and b) absent and/or weak response to isoagglutinins or vaccines. The antibody response was assessed by serology for viral infections and antipneumococcal antibody production. B and T cell counts were performed by flow cytometry. Secondary causes affecting immunoglobulin levels were also recorded. After the diagnosis, immunoglobulin infusion was initiated at doses between 466 and 600 mg·kg^-1^·month^-1^.

## Results

Eight patients (5 M and 3 F) with the diagnosis of hypogammaglobulinemia out of 350 PID patients were included. Patients undergoing their first visit were aged between 16 and 65 years (median: 41 years). The median age for symptom onset was 25 years (1 to 59) and diagnosis was established after 10 years. The history of early death of children of relatives was positive in 2/8 (25%) of the evaluated patients. There were reports of consanguinity (first cousins) in 3/8 (37.5%) patients, two of whom mentioned a family history of premature death. CVI was defined for 6 patients, transient hypogammaglobulinemia was defined in one adult (patient 1), and hypogammaglobulinemia after chemotherapy in one patient.

**Figure 1.**
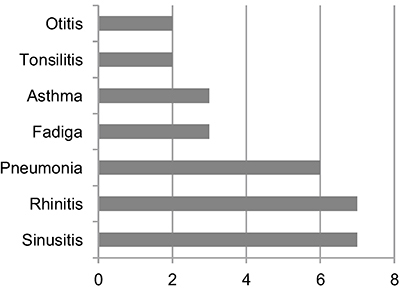
Clinical manifestations in the eight patients with hypogammaglobulinemia.

The most prevalent infections were sinusitis in 7/8 (87.5%), pneumonia in 6/8 (75%), otitis in 2/8 (25%), tonsillitis in 2/8 (25%), diarrhea in 2/8 (25%), and fatigue in 3/8 (37.5%) patients. Hypothyroidism was diagnosed in 4/8 (50%) of the patients. Allergic symptoms occurred in 7/8 (87.5%) patients who had allergic rhinitis and 3/8 (37.5%) who had asthma (Figure 1).

**Figure 2.**
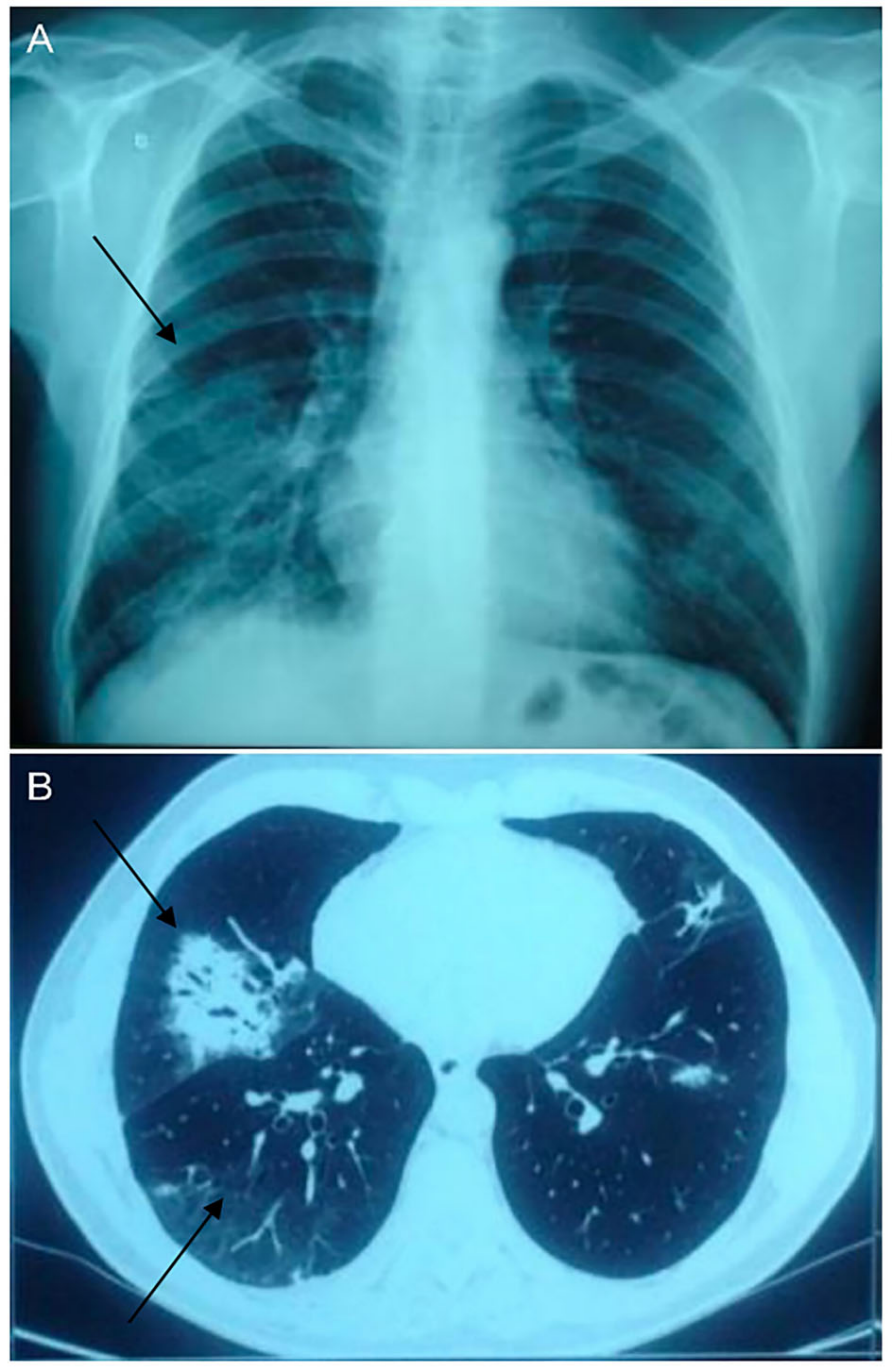
Pulmonary images of patients with hypogammaglobulinemia. **A**, Thoracic radiography performed during the first episode of pneumonia. **B**, Thoracic tomography performed during the first episode of pneumonia, evidencing multiple consolidations in the pulmonary lobes. The arrows indicate the pulmonary areas affected.

**Figure 3.**
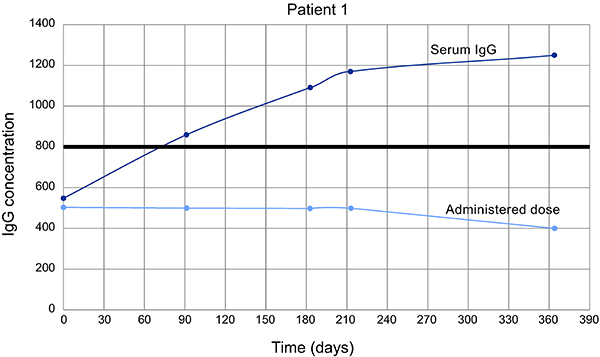
Levels of immunoglobulin G (IgG) and doses of intravenous immunoglobulin administered in a patient with hypogammaglobulinemia, showing later normalization of serum levels.

Patient 1, male, aged 65, was referred to the outpatient clinic after two episodes of pneumonia with identification of *Pneumocystis jirovecii*, where hypogammaglobulinemia and reduced TCD4+ cells were observed. This patient had not presented a history of previous infections. Intravenous immunoglobulin replacement was administered at a dose of 500 mg·kg^-1^·month^-1^ for one year. During follow-up, colonoscopy was performed as part of the routine examinations, and intestinal or colon adenocarcinoma *in situ* was detected and resected endoscopically. After the procedure, immunoglobulin levels rose slowly and a gradual withdrawal of intravenous immunoglobulin replacement was proposed. The patient maintained normal serum immunoglobulin levels and increased B cell numbers during the 3 full years of follow-up after discontinuation of therapy with immunoglobulin infusion (Figures 2 and 3).

Patient 7, female, presented uterine sarcoma at age 50 and underwent total hysterectomy followed by radiation therapy. At age 53, she was diagnosed with diffuse large B-cell lymphoma and then treated with chemotherapy. She was referred for immunological evaluation as a result of recurrent sinusitis every 2 months and chronic diarrhea. Once the diagnosis of secondary hypogammaglobulinemia had been made, the patient received intravenous immunoglobulin replacements with an average dose of 480 mg/kg. The other patients were diagnosed with CVI (Patients 2, 3, 4, 5, 6, and 8) (Supplementary Table S1).

All patients were submitted to chest computed tomography (CT) scans, which were normal in patient 7, who presented hypogammaglobulinemia after chemotherapy, and in patient 5, who was diagnosed with CVI. In the remaining patients, the following alterations were observed: atelectasis (3), bronchiectasis (2), opacity in ground glass (4), and budding tree (2). Bronchial inflammation was observed in 4 patients.

Administration of intravenous immunoglobulin was monitored in all patients and patient 6 was maintained with subcutaneous immunoglobulin with hyaluronidase. All patients, except the one who developed hypogammaglobulinemia after chemotherapy, received antibiotic prophylaxis.

## Discussion

Hypogammaglobulinemia may occur due to multiple causes. Of the primary immunodeficiencies, CVI is the most prevalent after IgA deficiency (1:1000 individuals) (11). In Brazil, a prevalence rate of 1:66,000–75,000 has been estimated (11). These data exhibit significant variability in several countries, likely due to healthcare accessibility, time to diagnosis, or even lack of patient identification. The genetic differences among the populations may also be relevant (4).

A European study with 2,212 patients reported that 1/3 of the patients manifested the disease before 10 years of age (6). The time to diagnosis in the present study was at least 10 years in half the population, longer than that observed in Europe or the United States (5,6,12). This aspect alone demonstrates the need to alert specialists in general to achieve the earliest possible diagnosis in Brazil.

Sinopulmonary infections (pneumonia, bronchitis, sinusitis, otitis, and conjunctivitis) by encapsulated bacteria and gastrointestinal infections (*Giardia lamblia* diarrhea) are the most common clinical manifestations (13,14). Although bacterial infections are characteristic of humoral immunity defects, Sperlich et al. (15) identified viral infection in 30 (56%) of 54 nasopharyngeal swabs collected in 41 immunodeficient patients. Rhinoviruses were most commonly detected (33%), but one of the patients presented metapneumovirus infection similar to one of the patients in our outpatient clinic (15).

The *Pneumocystis jirovecii* infection found in one of our patients with no previous history of infections, was unusual. This infectious agent is more commonly associated with hyper-IgM syndrome (16). Although the immunoglobulin profile of the patient did not suggest this diagnosis, the identification of *P. jirovecii* led us to evaluate the expression of CD40L, which was normal. During routine exams, *in situ* adenocarcinoma was detected and resected. There was regression of hypogammaglobulinemia, as evidenced by increasing IgG levels during pre-infusion monitoring. In the literature, there is only one description of hypogammaglobulinemia associated with renal cell carcinoma with resolution of the immune defect after resection of the tumor, similar to what occurred with our patient (17,18).

Chronic lung diseases are an important cause of recurrent hospitalizations, worse morbidity, and mortality. About 30 to 60% of CVI patients develop chronic lung disease, and bronchiectasis is more commonly found in 17 to 76% of patients (19,20). Of the 8 patients we followed, only two had pulmonary alterations in the first year of disease, as seen by chest tomography. On the other hand, two patients did not have alterations even after 7–15 years of disease, and the others established sequelae that occurred between 2 and 7 years after diagnosis.

Regarding tomographic findings, two patients, including one with hypogammaglobulinemia due to chemotherapy, had no sequelae although they had respiratory infections. This is the best time to initiate a therapeutic approach with immunoglobulins and reduce pulmonary sequelae.

Inflammation of the airways is common in CVI and may progress over time to obstructive or restrictive disease, bronchiectasis, atelectasis, and fibrosis, as evidenced by CT. The establishment of sequelae is also seen in a large number of patients who use prophylactic antibiotics to initiate the specific therapy, persisting longer with the infection and consequently evolving to pulmonary disease (15).

An interstitial lung disease known as granulomatous lymphocytic interstitial lung disease (GLILD) is often found and has a higher mortality rate, occurring in at least 10–20% of patients with CVI. GLILD presents tomographic findings distinct from the usual airway abnormalities commonly associated with CVI: pulmonary micronodules, thoracic lymphadenopathy, interlobular septal thickening, and multifocal pulmonary consolidation (21). Although atelectasis (3), bronchiectasis (2), ground glass opacity (4), and budding tree (2) were found, the criteria for granulomatosis diagnosis were not met by our patients.

Hypothyroidism was diagnosed in 4 of our patients. Quinti et al. (12) reported autoimmunity in 17% of 224 patients and, in 2.3%, this was the only manifestation at the time of diagnosis. Many autoimmune diseases have been described in the course of hypogammaglobulinemia, such as cytopenias, inflammatory bowel disease, seronegative arthritis, Sjögren's syndrome, uveitis, vasculitis, and vitiligo (22,23).

Agondi et al. (24), in Brazil, reported that allergic diseases are uncommon in CVI; however, rhinitis and asthma were observed more frequently in our study. Their diagnosis is somewhat difficult, since pulmonary lesions can result in symptoms similar to those of asthma.

Once patients are identified with serum immunoglobulin G (IgG) below the normal limit, the need for immunoglobulin replacement can be established. However, the finding of low IgG concentration itself, without a clinical manifestation, is still a subject of discussion regarding the appropriate treatment (25). In our study, there was marked variability in IgG levels at the time of diagnosis, from 30 to 563 mg/dL. These concentrations did not correlate with the severity of the clinical picture. Recently, the outpatient clinic evaluated a patient with reduced IgG serum levels, but without any clinical complaints. Thus, the patient was not included as CVI and treatment was not initiated (data not shown) showing that the antibody response to stimuli is more relevant than serum immunoglobulin levels. Robinson et al. (2008) found that 73% of children with CVI maintained normal isoagglutinin titers and 44 to 62% responded to protein antigens at the time of diagnosis. However, the response to pneumococci was impaired in the majority of patients (26). Driessen et al. (27) observed that in 44 patients with CVI and 21 with idiopathic primary hypogammaglobulinemia, both groups had severe pneumonias and bronchiectasis, although in the second group, IgG levels were only moderately reduced compared to CVI. Thus, half of the patients with idiopathic primary hypogammaglobulinemia required immunoglobulin replacement (27).

In 3 of 5 CVI patients in our study, B cell count was below 2.5%. Chapel et al. (28), in a retrospective review comprising mainly adults, found that 54% of the patients presented normal percentages (6–16%) of circulating B cells, 19% had elevated levels (>17%), 12% had reduced levels (1 to 6%), and 12% had undetectable levels (>1%).

Immunoglobulin replacement is the basis of treatment for hypogammaglobulinemia, reducing sequelae from the respective immunological defect (4,25). The treatment can be administered intravenously or subcutaneously with variable doses, as determined by an individualized orientation. The dose prescribed by the investigators ranged from 466 to 600 mg/kg per month, with good clinical response and serum IgG levels in 5 patients. The route of administration had to be changed from intravenous to subcutaneous in one patient due to adverse effects and in another patient due to the need for a higher dosage to reach adequate levels. After the review of immunoglobulin concentrations, patient 1 stopped receiving immunoglobulin replacement as previously described.

In the present study, one of the patients presented hypogammaglobulinemia secondary to chemotherapy. A study in patients with oncological diseases found that the monitoring of immunoglobulin levels is not established by specialists following chemotherapy. Even temporarily, reduced humoral response may cause recurrent infections in these patients and impair their quality of life (29).

The prognosis of the disease depends on the clinical phenotype of the patient. In situations with unexplained enteropathy, chronic lung disease, polyclonal lymphoproliferation, or cytopenia, survival may be lower. Clinical follow-up studies over four decades have found that the causes of death were respiratory failure of chronic lung disease (35%), lymphomas (18%), and neoplasms other than lymphomas in 10% to 33% (4).

Although we are presenting this report based on a restricted number of patients due to the short period evaluated and the recently established outpatient clinic, the cases illustrated clinical practices, demonstrating that hypogammaglobulinemia was associated with several causes and was presented in various ways. The diagnostic approach should be broad so that treatment could start early and frequent complications could be recognized.

## Supplementary Material

Click here to view [pdf].
